# Supplementary figures and images for: Effect of homophily and correlation of beliefs on COVID-19 and general infectious disease outbreaks
Source: PLoS One. 2021 Dec 2;16(12):e0260973. doi: 10.1371/journal.pone.0260973 (PMC8639064; doi:10.1371/journal.pone.0260973)

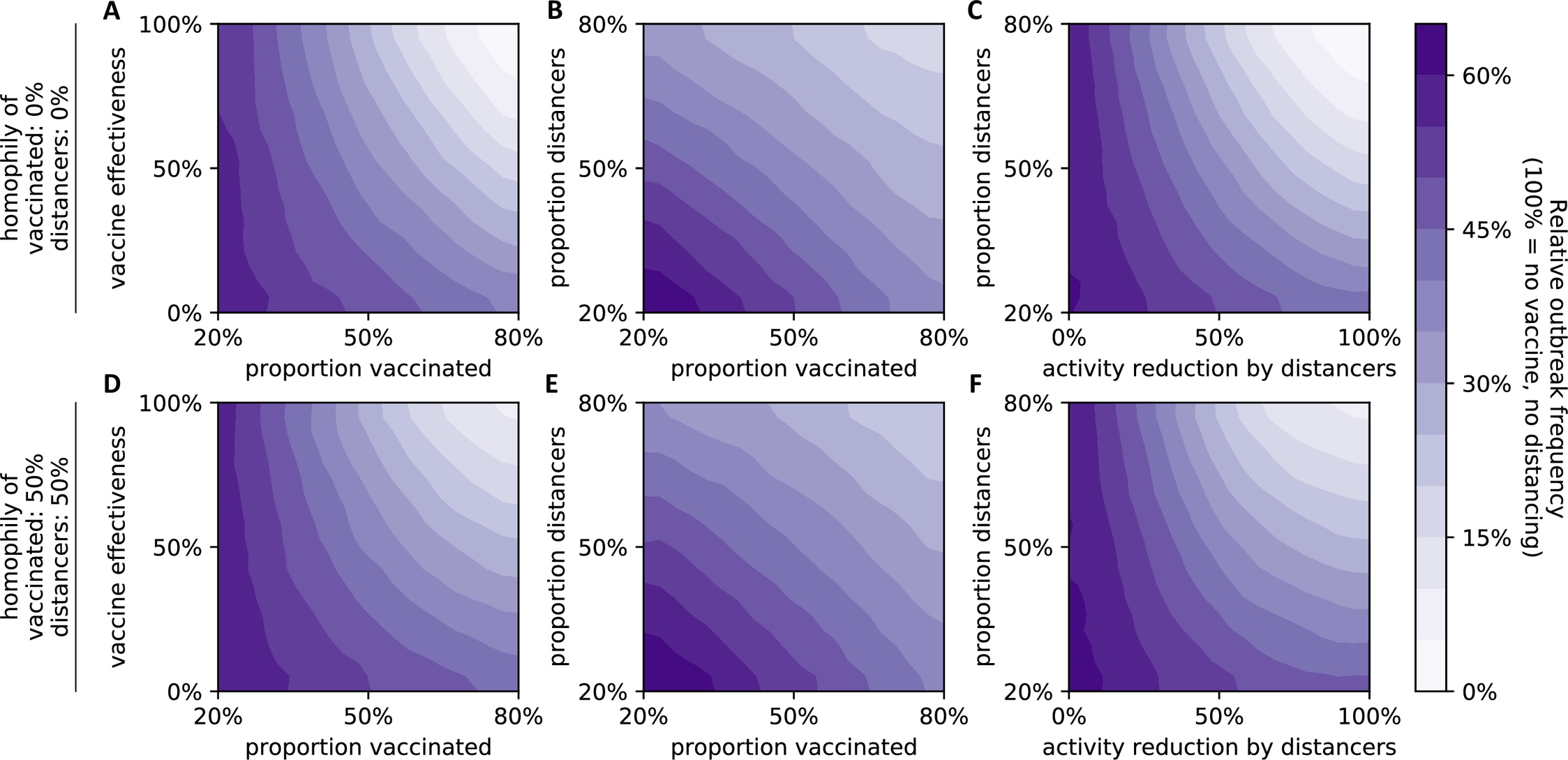

Supplement: S1 Fig — Contour plots were generated from 10,000,000 independent simulation runs with four vaccine and social distancing parameters chosen uniformly at random (axes show parameter ranges). The outbreak frequency Black (where an outbreak was defined as >1% of the population eventually becoming infected) from a reference scenario of no vaccine and no social distancing was calculated for two scenarios: Black (A-C) social interaction networks with 50% homophily of those who vaccinate and of those who practice distancing and (D-F) networks without homophily. Data was binned and smoothed using a two-dimensional Savitzky-Golay filter [18] (details in Methods). Each subplot shows the effect of variation of two parameters on the relative outbreak frequency. (A,D) vaccine coverage (x-axis) and vaccine effectiveness (y-axis), (B,E) vaccine coverage (x-axis) and proportion of those who distance, (C,F) contact reduction (in %) by those who practice social distancing (x-axis) and proportion of those who distance (y-axis). A comparison of the outbreak frequency under the two scenarios is shown in Fig 2, an equivalent analysis for the basic reproductive number in S2 Fig. (TIF) [file pone.0260973.s001.tif]

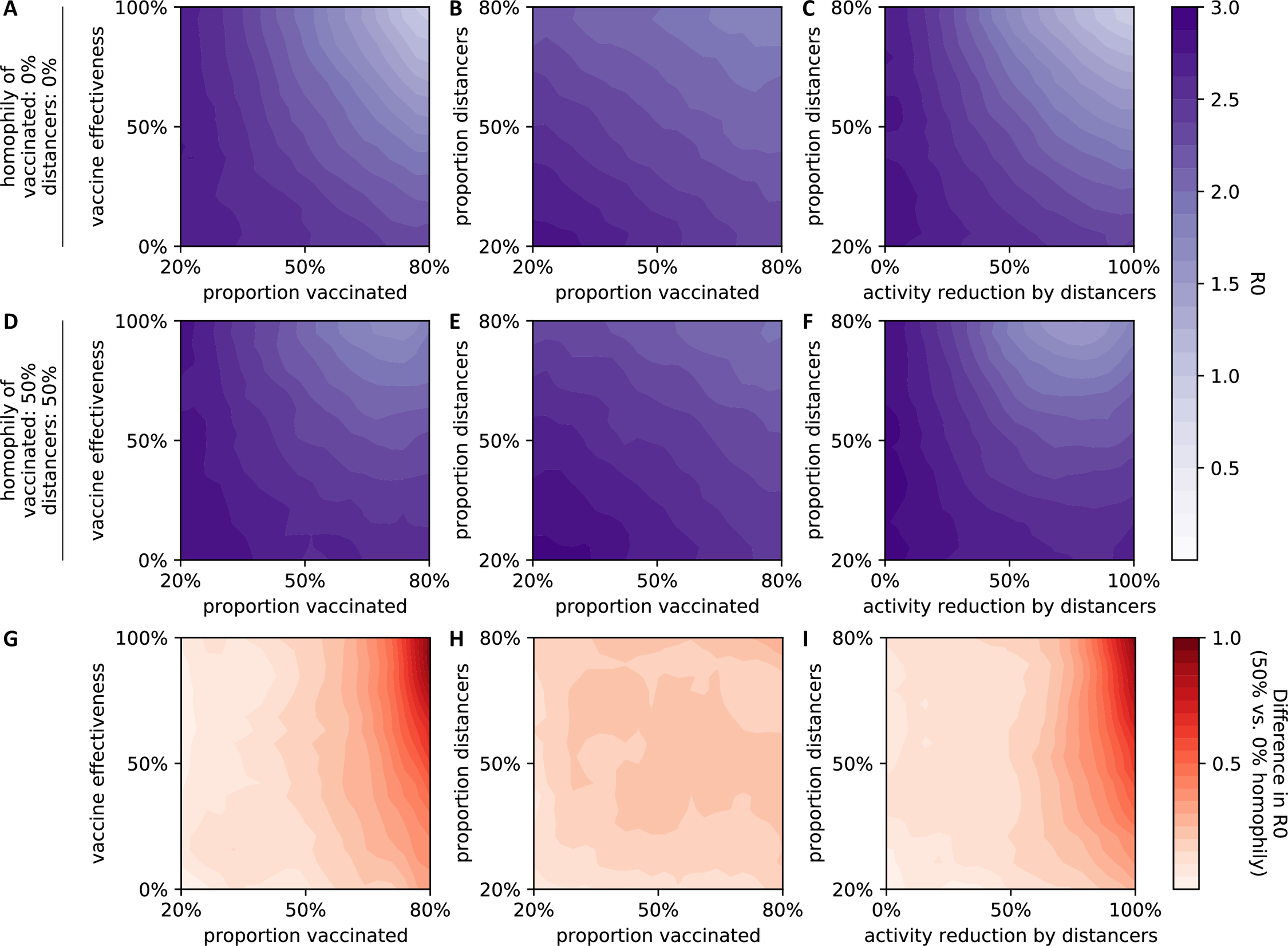

Supplement: S2 Fig — Contour plots were generated from 10,000,000 independent simulation runs with four vaccine and social distancing parameters chosen uniformly at random (axes show parameter ranges). The basic reproductive number is shown for (A-C) social interaction networks with 50% homophily of those who vaccinate and of those who practice distancing and (D-F) networks without homophily. Data was binned and smoothed using a two-dimensional Savitzky-Golay filter [18] (details in Methods). (G-I) Comparison of the basic reproductive number in networks with and without homophily. Each subplot shows the effect of variation of two parameters on the basic reproductive number (A-F) or difference thereof between the two scenarios(G-I): (A,D,G) vaccine coverage (x-axis) and vaccine effectiveness (y-axis), (B,E,H) vaccine coverage (x-axis) and proportion of those who distance, (C,F,I) contact reduction (in %) by those who practice social distancing (x-axis) and proportion of those who distance (y-axis). (TIF) [file pone.0260973.s002.tif]

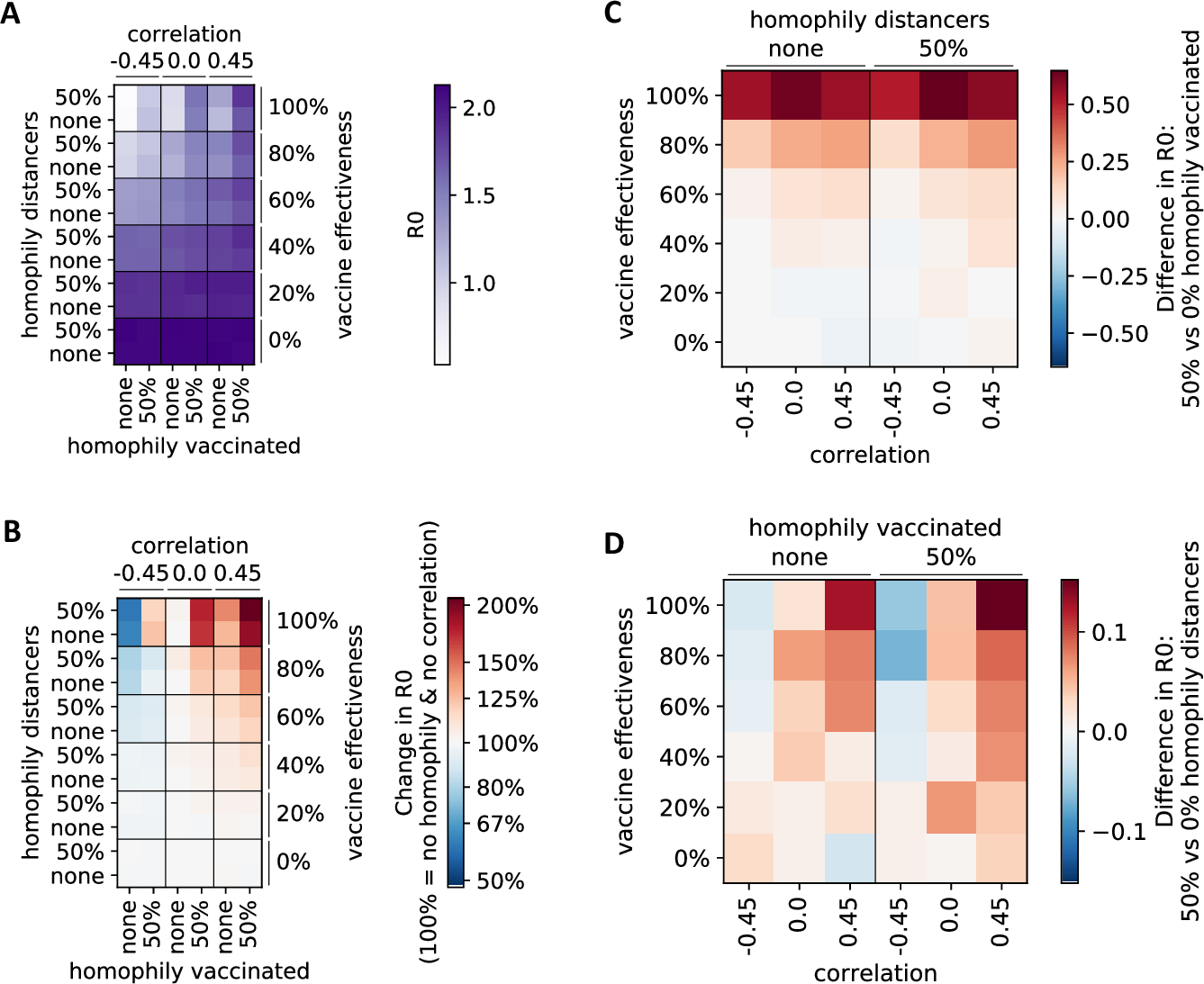

Supplement: S3 Fig — (A) The basic reproductive number (R0) is compared for different scenarios regarding homophily and correlation of those who vaccinate and those who distance, and for different levels of vaccine effectiveness. (B) For each level of vaccine effectiveness, the change in R0 is compared to the homogeneous case of no homophily and no correlation, which is set to 100%, respectively. (C-D) Absolute difference in R0 (from A) when comparing physical interaction networks where (C) vaccinated, (D) distancers cluster (homophily = 50%) versus networks without homophily. (TIF) [file pone.0260973.s003.tif]

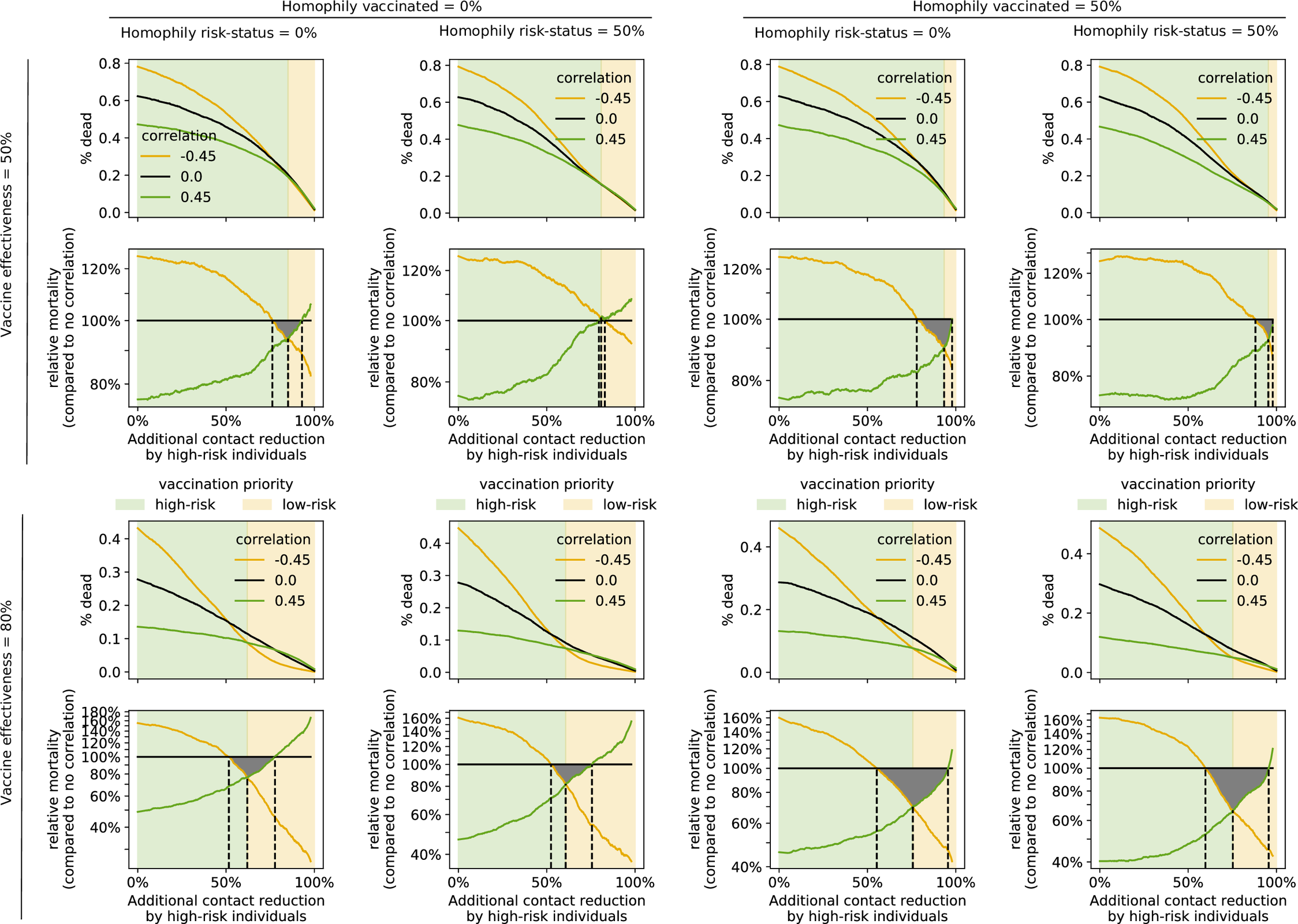

Supplement: S4 Fig — The average absolute mortality (first and third row) at a given additional contact reduction by high-risk individuals is shown for three different scenarios: negative (−0.45; yellow), zero (black) and positive (0.45; green) correlation between vaccinated and high-risk individuals. In addition, the relative mortality compared to the case of no correlation (black line) is shown (second and last row). Black dashed lines and a gray triangle highlight the three intersection points of the three curves. Different situations are considered: 50% (first two rows) vs 80% (last two rows) vaccine effectiveness, 0% (first two columns) vs 50% (last two columns) homophily of those who vaccinate, and 0% (first and third column) vs 50% (second and last column) homophily of high-risk individuals. For all eight scenarios, a direct comparison of the location of the gray region in between the intersection points is shown in Fig 6C. (TIF) [file pone.0260973.s004.tif]

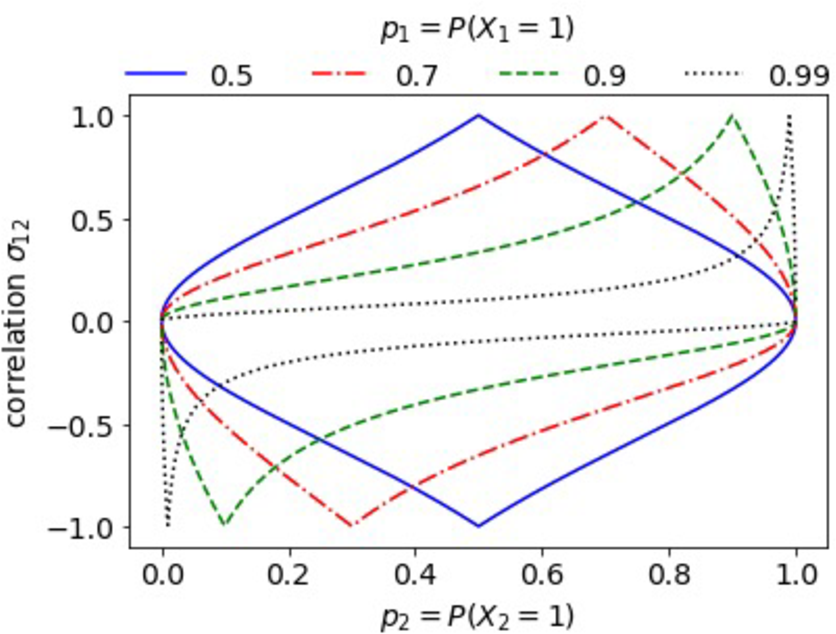

Supplement: S5 Fig — The possible range of correlations between two Bernoulli random variables with expectations p1 (colors) and p2 (x-axis) is shown for four fixed choices of p1. (TIF) [file pone.0260973.s005.tif]

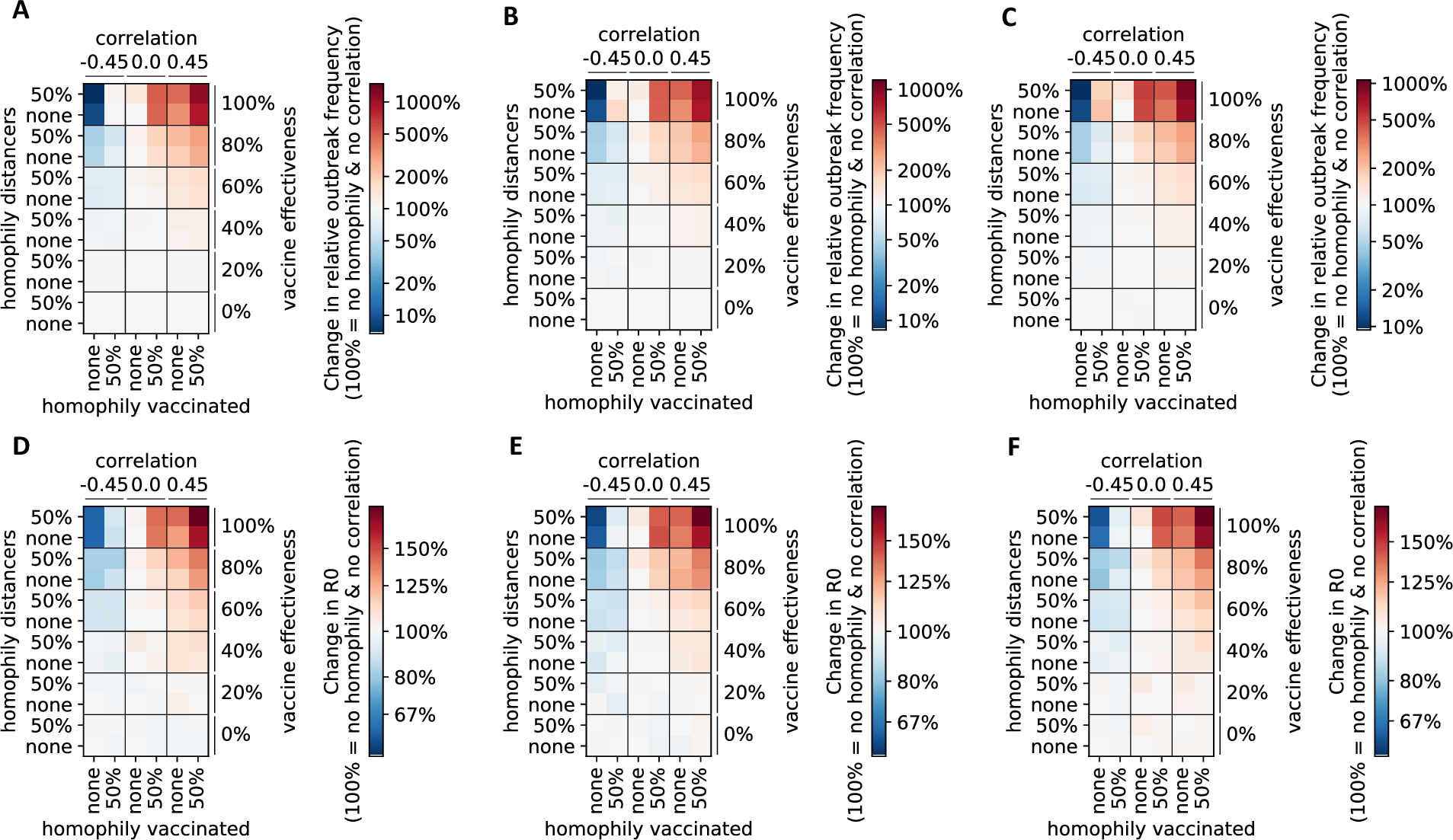

Supplement: S6 Fig — The change in (A-C) relative outbreak frequency and (D-F) basic reproductive number R0 compared to the homogeneous case of no homophily and no correlation is shown for different scenarios regarding clustering and correlation of those who vaccinate and those who distance, as well as for different levels of vaccine effectiveness. The exponent used in the homophily algorithm (see Methods) is 1 in A and D, 4 in B and E, and 16 in C and F. (TIF) [file pone.0260973.s006.tif]

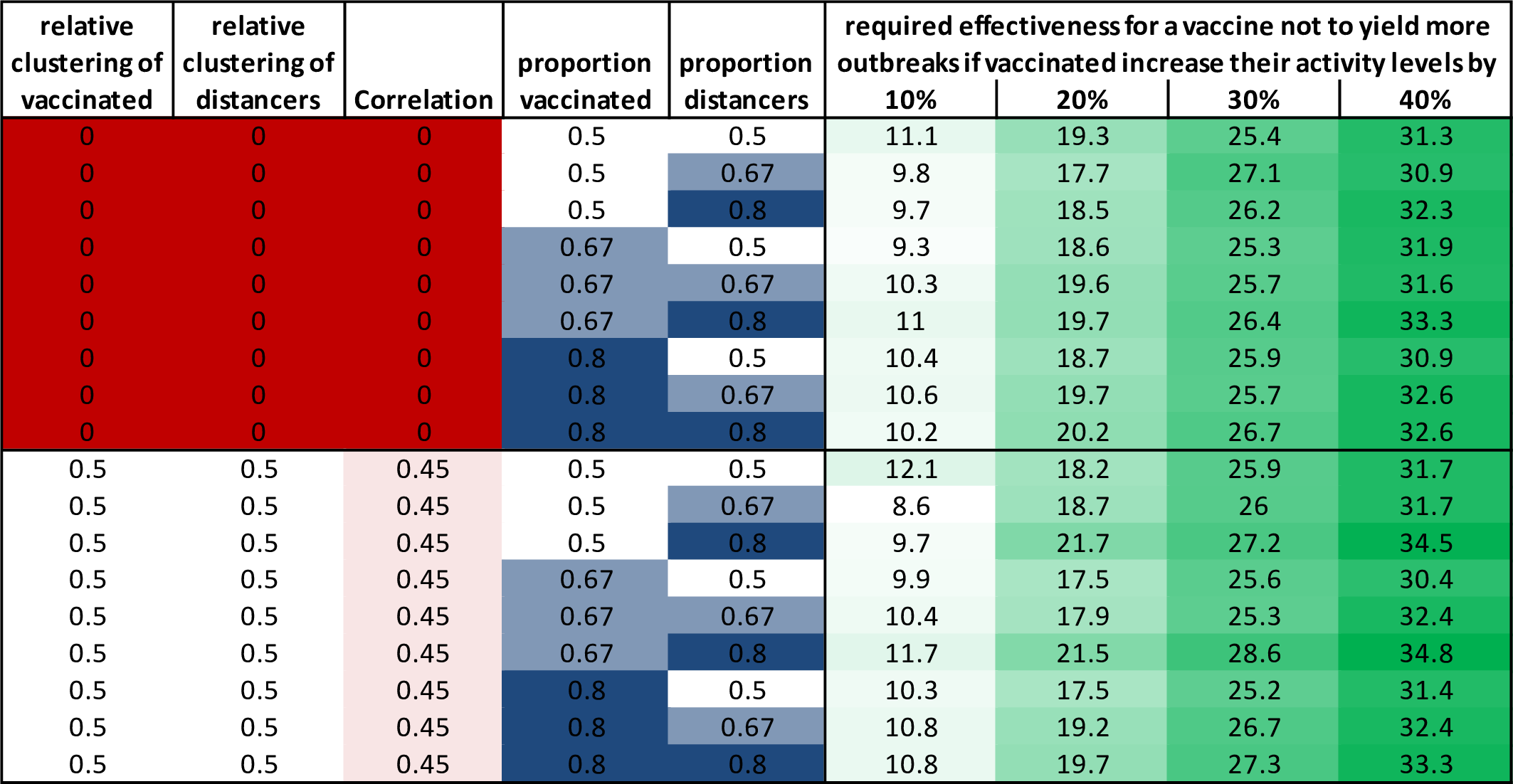

Supplement: S1 Table — For different proportions of those who vaccinate and those who distance (50%, 65%, 80%) and two scenarios regarding homophily and correlation (none versus high homophily and correlation), N = 200, 000 simulations were conducted for each considered level of increased activity by those who vaccinated (10%, 20%, 30%, 40%) with randomly chosen vaccine effectiveness, U([0%, 100%]), in addition to 200, 000 simulations each without a vaccine. Using a one-dimensional Savitzky-Golay filter with window size 20, 000 and linear functions, we obtained smoothed plots of the outbreak probability against the vaccine effectiveness for each increased activity level by vaccinated, and inferred the respective vaccine effectiveness (green cell values) at which the outbreak frequency under scenarios with a vaccine and increased activity levels by the vaccinated equaled the outbreak frequency without a vaccine (see the black line in Fig 4 for an example). (TIF) [file pone.0260973.s007.tif]

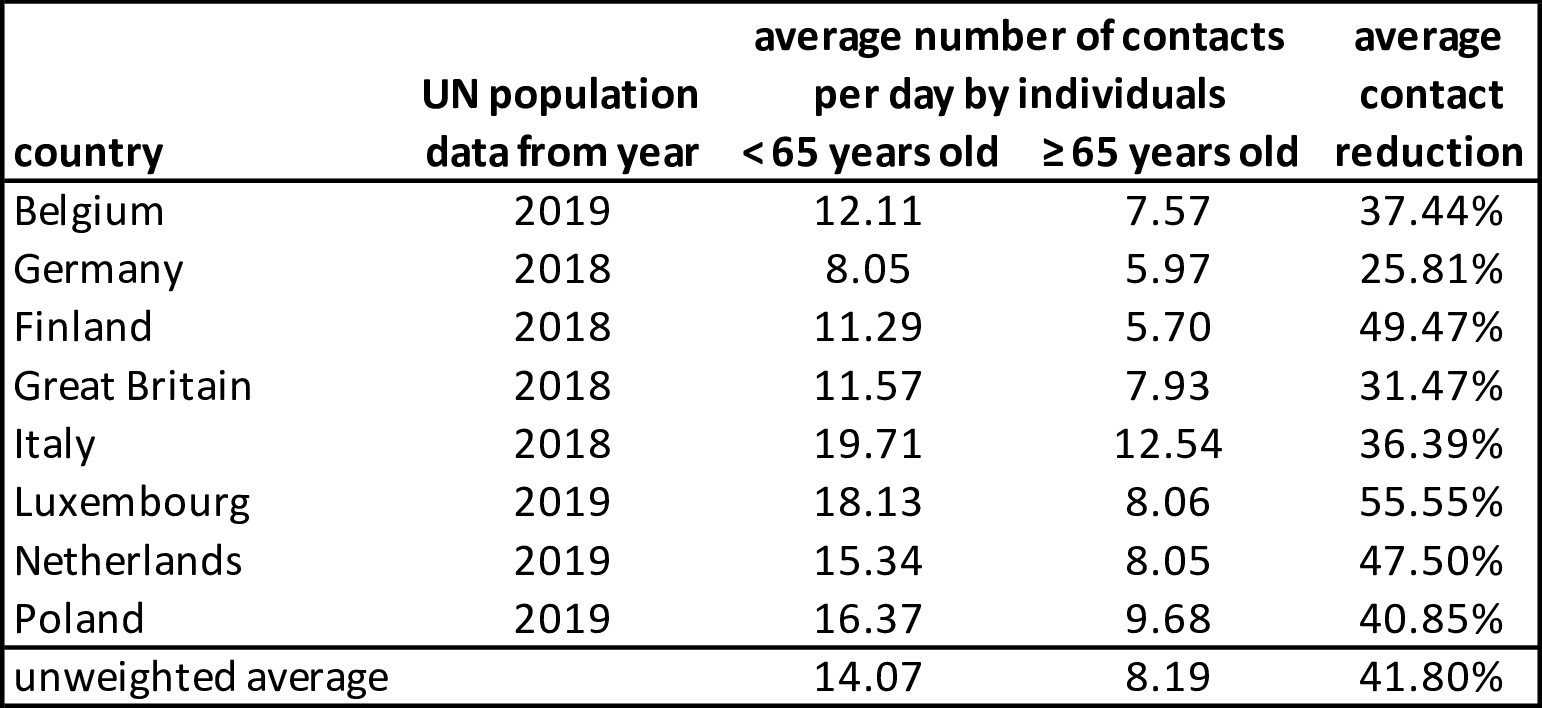

Supplement: S2 Table — Data from [21]. The most recently available census estimate from the United Nations Demographic Statistic Database was used for a weighted average of the contact rate across different age groups. The average contact reduction (last column) is calculated as one minus the ratio of average daily contacts by older people (fourth column) over the average daily contacts by younger people (third column). (TIF) [file pone.0260973.s008.tif]
