# Supplementary material for: Effect of homophily and correlation of beliefs on COVID-19 and general infectious disease outbreaks
Source: PLoS One. 2021 Dec 2;16(12):e0260973. doi: 10.1371/journal.pone.0260973 (PMC8639064; doi:10.1371/journal.pone.0260973)
Supplement: Attachment — Submitted filename: response_to_reviewer_final.pdf [file pone.0260973.s010.pdf]

**Response to reviewer comments**

Reviewer #1:
I was happy to review this paper again, being Reviewer #1 for the previous submission. In my opinion, the paper has improved in many respects, especially regarding the clarification of some terms and definitions (especially in the title), the presentations of results, and the discussion of the modeling decisions. I want to thank the authors for their careful consideration of my comments. While most of them have been addressed, there is one comment that I would like to discuss again. I also added a few minor points. Finally, I understand the authors' wish to keep a high level of quantitative detail in the figures. Nevertheless, I took the liberty of adding a few suggestions on the figures, in case they still wish to improve their readability.

We thank the reviewer for this generally positive assessment of our study and our revisions. Below, we describe how we addressed the additional comments and concerns. Most edits (unless full paragraphs were added) to the manuscript are mentioned in here and shown in red.

Major comment:
1. Following my previous major comment #3, I still believe the use of the term "outbreak probability" in the paper is misleading, because the authors only consider simulations starting with susceptible seed nodes; The probability they compute is in fact a conditional probability, conditional on the seed being susceptible, which is an important point to mention and explain. In any case, I still believe it would be better to consider the probability of having an outbreak when a random individual gets in contact with the virus, without conditioning on the seed being susceptible, mainly for two reasons.
First, I believe the second probability makes more sense for the problem at hand and the readers the authors want to address. Let us imagine the network being a community on an island, on which the virus arrives (maybe through a tourist visiting). I believe the authorities on the island will rather ask how likely they will have to manage an outbreak given that the virus arrived, rather than how likely they will have to manage an outbreak given that the virus arrived and that it reached a non-protected individual. Indeed, the scenarios in which the virus reaches a protected individual and nothing happens will matter to them, if there are many of them, they might deem allowing tourists safe enough.
Second, it is meaningless to compare conditional probabilities when conditions are different. Conditioning simulations on seeds being susceptible means that the number of outbreaks between simulations with different levels of vaccine effectiveness cannot really be compared. This is of course because simulations with more effective vaccines are more likely to start with anti-vaxxers, and if social distancing is correlated to vaccinating, these individuals are more likely to be sources of outbreaks.
Choosing to condition on the seed being susceptible or not probably does not affect most results in the paper, as the values examined are mostly changes in outbreak probabilities, or relative probabilities, with the number of vaccinated people and the vaccine effectiveness being constant. Considering one or the other probability is just a matter of a multiplicative factor. However, it does seem to matter in Figure 4: There, the outbreak probability (the conditional one proposed by the authors) is shown in absolute values and compared across different values of vaccine effectiveness, which seems problematic to me.
I would suggest that the authors clarify their definition of outbreak probability and revisit their

results on increased activity levels on outbreak probability (Figure 4), as well as other comparisons of conditional probabilities shown in the supplementary materials (Figures S1 A and D, S2 A and D, and S3 A and B).

We agree with the reviewer. Our definition of outbreak probability is really a conditional probability. We addressed this issue by switching to a relative outbreak frequency, which we agree makes more sense for the two reasons mentioned by the reviewer. This significantly strengthens the interpretability of our study and we thank the reviewer for pushing us to make this change.

To calculate the relative outbreak frequency (without having to rerun any model simulations), we multiplied the original conditional outbreak probability with an appropriately chosen factor that is proportional to the total number of contacts by susceptible individuals in the respective scenario. Depending on the type of analysis, we set a different scenario as the reference scenario (e.g., no vaccine and no distancing in Figure 2 and corresponding S1 Fig, or no homophily and no correlation for each level of vaccine effectiveness in Fig 3B). For each figure, we include a statement such as "100% = no vaccine" in the label of the gradient scale and explain in the legend. We added detailed methods explaining this calculation in the Methods section "Outcome measures". Rather than copying all the added text, we refer the reviewer to this subsection.

As part of this change, we also decided to move S4 Fig A back into Fig 3. It shows the relative outbreak frequency for different choices of homophily, correlation and vaccine effectiveness and is used to compute the changes and differences in relative outbreak frequency shown in the rest of the figure.

Further, we would like to point out that the change to relative outbreak frequency also got rid of the model artifacts that we observed for the conditional outbreak frequency, described in the paragraph in the Discussion starting with "Perfect isolation by those who practice social distancing". The artifacts are however still present when considering the basic reproductive number and we adapted the paragraph to account for this. It now reads as follows:

> Perfect isolation by those who practice social distancing led to higher R0 values than very high levels of distancing (S2 Fig, F). Similarly, in the case of a highly effective vaccine very high vaccine coverage (80%) led to more outbreaks than slightly lower coverage (S2 Fig, D). Both these counter-intuitive observations only occurred in the presence of homophily, and they are likely model artifacts: The activity level of each individual corresponds to the probability that this individual is chosen as the initially infected seed case. If the activity level of distancing individuals is nonzero (e.g. 75% reduction), then there remains a small chance that an individual who distances is chosen as the seed case. If this happens, passage of the virus is unlikely in the presence of homophily, i.e. when the distancers cluster together. At perfect isolation (100% reduction), solely non-distancers, who in the presence of homophily cluster together, are chosen as seed cases. Similar reasoning explains the second observation. Note that these counter-intuitive observations did not occur when considering the relative outbreak frequency as this measure, contrary to R0, takes into account the probability that an outbreak actually

> occurs. This is another reason why we used the outbreak frequency as the primary outcome measure in the generic infectious disease model.

Minor comments:
1. I was a bit surprised to notice that the term of “agent-based model” was never used - using the term might help some readers identifying more rapidly the nature of the models.

We have revised the introduction and methods to include a mention of the agent-based nature of the model:

> Lines 36-39 (Introduction): In this study, we present a novel technique for applying binary attributes with a pre-defined correlation structure to an agent-based physical interaction network that exhibits a pre-defined level of homophily for each attribute.
>
> Lines 43-45 (Introduction): First, we consider a simple compartmental infectious disease model ~~with~~ in which each agent has two binary belief attributes: confidence in vaccines and attitude toward social distancing measures.
>
> Lines 306-308 (Methods): We studied the spread of a generic infectious disease as well as COVID-19 throughout an agent-based physical interaction network of N = 1000 individuals, modeled as a Watts-Strogatz small-world network [16].

2. I fear my previous comment on discussing the influence of picking a node proportionally to its activity level (minor comment #12 previously) was misinterpreted. I did not mean to suggest that the model specification should be altered but that the consequence of this decision could be explained to the reader, if possible (without this assumption, would current results be stronger, weaker, the same…?).

The alternative to picking a seed node with a probability proportional to its activity level would be to choose a seed node completely at random. We believe this is less realistic than the current model specification and therefore chose not to discuss this scenario in the manuscript.

Hypothetically, in a scenario without a vaccine-induced increase in activity levels, all agents socialize either at a mean rate or at a reduced rate because of social distancing measures. In this case, choosing a seed case completely at random would reduce the probability of an outbreak because more social distancers would be chosen as the seed, and the infection would therefore have a smaller chance of taking off. This effect would be stronger in situations with a high level of social-distancing homophily. The scenario with both a vaccine-induced increase in activity and social distancers is more complicated, and the results would depend on both the relative proportions of socializing vaccinated (plus vaccine effectiveness) and social distancers, as well as the degree of increased and decreased activity of these two populations. We would have to run the model to determine exactly what effect changing the seed case to completely-at-random would have on disease outcomes in this scenario.

3. In the description of Figure 1, the phrase “removal of those successfully vaccinated” might

still be misleading for those unfamiliar with epidemiological models, maybe simply add “from the pool of susceptible individuals”?

We followed the reviewer’s advice and added “from the pool of susceptible individuals”.

4. In the description of Figure 2, the subparts A, B, and C are not mentioned.

We thank the reviewer for this observation. The new legend now reads (added parts in red):

> Fig 2. **Comparison of outbreak frequency in networks with and without homophily**. Contour plots were generated from 10,000,000 independent simulation runs with four vaccine and social distancing parameters chosen uniformly at random (axes show parameter ranges). The difference in outbreak frequency (where an outbreak was defined as > 1% of the population eventually becoming infected) from a reference scenario of no vaccine and no social distancing was calculated for two scenarios: social interaction networks with 50% homophily of those who vaccinate and of those who practice distancing and networks without homophily (see S1 Fig). Data was binned and smoothed using a two-dimensional Savitzky-Golay filter [18] (details in Methods). Each subplot shows the effect of variation of two parameters on the difference in outbreak frequency between the two different homphilly scenarios (see S1 Fig). (A) vaccine coverage (x-axis) and vaccine effectiveness (y-axis), (B) vaccine coverage (x-axis) and proportion of those who distance, (C) contact reduction (in %) by those who practice social distancing (x-axis) and proportion of those who distance (y-axis). An equivalent analysis for the basic reproductive number is shown in S2 Fig.

5. In the description of Figure 2, there is a typo: “¿1%”.

We fixed the typo. It now reads: >1%.

6. The phrase “100% = no homophily & no correlation” in Figure 3a is unclear.

To clarify what we mean by “100% = no homophily & no correlation” we added the following in the figure legend (in red). (Note that we also decided to move S4 FigA to Fig 3A to better highlight how the relative outbreak frequency (major comment) shown in Fig 3C,D (previously B,C) is calculated.)

> Effect of homophily and correlation of opinions on outbreak frequency. (A) The relative outbreak frequency is compared for different scenarios with respect to homophily and

correlation of those who vaccinate and those who distance, and for different levels of vaccine effectiveness. Reference level for comparisons is a vaccine with 0% effectiveness and no homophily nor correlation of vaccinated and distancers. This reference level is set to 100%. (B) For each level of vaccine effectiveness, the change in relative outbreak frequency is compared to the homogeneous case of no homophily and no correlation, which is set to 100%, respectively. (C-D) Absolute difference in relative outbreak frequency (from A) when comparing physical interaction networks where (C) vaccinated, (D) distancers cluster (homophily = 50%) versus networks without homophily.

7. There are a few places in the text where the term "Figure" appears without a number.

We apologize for this mistake. These issues stem from wrong uses of the Latex commands \ref and \nameref. We fixed it.

Suggestions/notes about the figures:
1. The use of red for decreased mortality and blue for increased mortality in Figures 3 and 5 might be unusual, some readers might interpret these colors the other way around (red often being negatively connotated).

We thank the reviewer for this very good suggestion. We flipped the colormap in all figures that show mortality (Fig. 3 and 5). We also flipped the colormap in all other figures where we display the change in other outcome measures so that now red consistently denotes the negative and blue the positive outcome.

2. The understanding of the figures might be improved by using colors consistently across different plots regarding the values they display.

We improved the consistency of the colors by doing the following:

a) When displaying a change in outcome measure (outbreaks, R0 or mortality), we now always associate red with the negative and blue with the positive outcome (white = no change)
b) We use a consistent gradient scale (purple to white) to display the ``raw" outcome measures in Figures 2A, S1, S2, S3A.
c) We use completely different colors (green and dark yellow) in Fig. 6AB, S4 to distinguish different scenarios for the correlation between risk- and vaccine-status.
d) We changed the gradient scale in Fig. 4 from a sequential to a diverging colormap. This colormap is better suited to highlight the results of this analysis: to show the increase (red) or decrease (blue) in outbreaks following the introduction of a vaccine and subsequent increase in activity levels by the vaccinated.

3. Figure 4 could include a third plot where the black line shown in the first two plots (to indicate the switch between the detrimental and beneficial presence of a vaccine) is shown for different homophily/correlation situations, in order to compare them more easily.

For an earlier version of the manuscript, we had already created a plot as suggested by the reviewer. This plot showed the black lines from Fig 4 for 18 scenarios (2 scenarios: homophily & correlation vs no homophily and no correlation, 3 values for the proportion of people who vaccinate, 3 values for the proportion of people who distance). All lines were very similar, which is why we decided to rather display this sensitivity analysis in a table (Table S1). Note that the updated Table S1 (updated to be based on the relative outbreak frequency instead of the conditional outbreak probability) shows even more similar values across the 18 scenarios.

4. In Figure 3a and 5b and 5c, the label “vaccine effectiveness” might be mistaken for the label for the gradient scale.

We changed the location of the label of the gradient scale. It is now displayed to the left of the gradient scale to avoid any confusion.
