# Supplementary material for: Effect of homophily and correlation of beliefs on COVID-19 and general infectious disease outbreaks
Source: PLoS One. 2021 Dec 2;16(12):e0260973. doi: 10.1371/journal.pone.0260973 (PMC8639064; doi:10.1371/journal.pone.0260973)
Supplement: Attachment — Submitted filename: PLOS comp bio reviewer comments responses 210719.pdf [file pone.0260973.s009.pdf]

Reviewer's Responses to Questions

Reviewer #1: This paper presents an interesting use of agent-based modelling to understand the impact of homophily and correlations between individual attributes on the dynamics of Covid-19 infections. Overall, the idea is interesting, the analyses are thorough, and the results raise an important point for vaccination strategies, by showing that the clustering of similar individuals and opinions might mean that optimal vaccination strategies should not necessarily prioritize risk subjects. This being said, I noted some points that I feel could be improved in the current manuscript, particularly regarding the initialization of the simulations and the presentation of the results.

Major comments:
1. In the title, both the terms "clustering" and "correlation of belief systems" can be misleading. In network terms, clustering is often a purely structural description of a system (i.e., individuals form tight communities). It should be clear that the analyses are not about structural clustering, but rather about homophily (i.e., the clustering of similar individuals in the network). Regarding the correlation of belief systems, I am not sure whether this term is used in this sense among social scientists; "correlation of beliefs" or "belief consistency" might be more appropriate (see "Measuring Belief System Structure" from Barton & Parsons, 1977). In any case, the paper examines the correlation between social distancing behavior, willingness to vaccinate, and being a risk subject; so, the third aspect is not a belief anyway. I would suggest changing these two terms.

We have revised our language throughout the paper, replacing "clustering" and "relative clustering" with "homophily," and "correlation of belief systems" with "correlation of beliefs or circumstances" or simply "correlation of beliefs".

We have also revised the Introduction (line 22) to clarify what we mean by the correlation of beliefs:

> Especially during a pandemic, when risk mitigation measures are implemented globally, homophily regarding various beliefs <u>or circumstances</u> and <u>population-wide</u> correlations between them may have profound effects on disease spread.

2. The structure of the network could play a role in the analysis of the model, and especially the level of structural clustering in the network. It could be possible to have a network with loosely connected communities that are very homophilous, and another one with tight and segregated communities that are a less homophilous. The dynamics of the disease might be different in these two networks, and this effect could be missed in simulations only using small-world networks with a fixed rewiring parameter. I would recommend at least discussing the role of the network structure, and potentially doing some robustness checks varying the amount of structural clustering. Other types of networks could be used for that (see Hamill, L. & Gilbert, 2009 "Social circles: a simple structure for agent-based social network models", for example).

We agree with the reviewer and thank the reviewer for this important clarification. We have added the following paragraph to the Discussion, following the reviewer's suggestion.

> Homophily, the main property studied in this paper, is as a type of clustering that is based on node characteristics. Small-world networks such as those used in this study also exhibit a more traditional type of ``structural" clustering, measured using e.g. the local clustering coefficient (Watts and Strogatz 1998, Hamill and Gilbert 2009). This type of clustering captures the idea that friends of a friend are likely to be friends and is based on edge characteristics. Most nodes in a small-world network cannot be assigned to a single cluster as there exists high levels of overlap among the clusters. It would be interesting to explore how the presence of homophily affects the spread of disease through social interaction networks with more defined structural clusters, e.g., mimicking the separation of a community into discrete sub-communities.

3. In my understanding, simulations are started by picking a node that is not vaccinated. This can be an important modelling decision that makes the comparison of different scenarios with different vaccine effectiveness difficult. Indeed, a better vaccine means more social-distancing nodes taken out and more non-distancers left out to start with (and potentially better candidates to start an outbreak). In other words, that decision could artificially inflate the probability of an outbreak. If that is the case, it would be better to include in the calculation of the probability of an outbreak the simulations that "never started" because a vaccinated node was the seed, since there is no reason to expect the virus to select non-vaccinated individuals. In any case, this part of the model should be discussed.

We thank the reviewer for pointing out a possible confusion. Simulations are started by picking a node that is not **successfully** vaccinated (i.e., a susceptible node). As we state in the Methods section (line 448): "The simulation starts with a single seed case: a susceptible who becomes infected. […] The probability that any susceptible becomes the seed case is proportional to the activity level of that individual." Unvaccinated individuals and unsuccessfully vaccinated individuals make up the pool of susceptibles (note we use an all-or-nothing vaccine as stated in the Methods and Discussion and now also in a revised caption to Figure 1). Assuming, as suggested by the reviewer, that all vaccinated individuals (including those successfully vaccinated) should be included as potential seed cases does not make sense for two reasons. First, successful vaccination prevents infection and—to at least a large degree—spread of the virus. Second, we only compare scenarios where the vaccination coverage is fixed (except in the first (validation) analysis reported in Figure 2). We made this choice because we are interested in exploring how the distribution of the vaccinated across the network affects disease dynamics. Inclusion of simulations that "never started" because a vaccinated node was the seed would thus not change the relative results. We would see the same reduction in all scenarios with the same vaccination coverage.

We do however agree with the reviewer that part of the results may be “intuitively explained” by understanding the way the model is implemented and works. Relative differences in epidemic outcomes increase in the presence of
(i) a better vaccine, and
(ii) a positive correlation between vaccinated and distancers, and
(iii) positive homophily w.r.t. both attributes
partly because the simulation in this particular setting is more likely to start within a cluster of non-vaccinated non-distancing individuals. But does the fact that there exists an explanation for a finding make the finding irrelevant? We are certain that most if not all findings made in this modeling study and in other similar studies can be understood through a detailed study of the model itself. That does not make the findings irrelevant, especially to the general public and public policy makers who often lack the required modeling expertise.

4. Since the model is a relatively simple ABM, with a number of parameters kept low to ensure interpretation, precise quantitative interpretation of the simulations does not seem very important because they most likely will not match reality. It seems much more interesting instead to focus on the qualitative behavior of the model. This is what the text does mostly, but my impression is that the figures (2 to 6) show an unnecessary amount of information and quantitative results, which hinders the reading and the interpretation of the model. Although interesting in themselves, some of these plots could be used as supporting information. Simpler visualizations of the main results of the paper would be much more useful in the main text. For example, having one plot per result would help rather than sometimes referring to a series of plots to illustrate a single point in the text (see lines 67, 68 for example). Importantly, any variation on the parameters that does not affect the interpretation of the model should probably be presented as a robustness check in the appendices.

We agree with the reviewer. Precise, quantitative predictions cannot be produced by our ABM. This is however true for most if not all studies of epidemics on networks and was also not the goal of this study. The goal was to exhibit how the presence/absence of homophily in beliefs and circumstances influences the spread of a virus.

We slightly disagree with the reviewer that Figures 2 to 6 show an unnecessary amount of detail. Alternative figures which simply show qualitative measurements would not constitute good science in our opinion. The model was used to produce quantitative predictions, which we show as they were produced. As mentioned above, we agree that the exact quantitative values may be off but that is why we have interpreted the results qualitatively and not quantitatively in both the Results and the Discussion (see also our response to major comment 5).

As suggested by the reviewer, we have simplified some of the visualizations. We have produced a new Figure 2, which explicitly shows the absolute difference in outbreak probability when comparing the 50% with the 0% homophily scenario. This simplifies the story-telling as the point made in the text is immediately visible form the new figure.

Moreover, we followed the reviewer's advice and moved a less important subplot (panel A of Figure 3), as well as the complicated original Figure 2 into the supplement.

5. The discussion on vaccination strategies will probably the most interesting part for many readers. I would therefore recommend that the authors explain in more detail what they mean by "prioritizing" individuals. Is this prioritization meant in a dynamic way (i.e., if doses of vaccines are received progressively and authorities have to decide in which order individuals get access to the vaccine), or is it meant in absolute terms (i.e., if the total number of doses is fixed to a certain percentage of the population and only some individuals can get the vaccine)? Does prioritizing high-risk individuals mean vaccinating all high-risk individuals and give the remaining vaccines to others, or does it simply mean having more high-risk individuals vaccinated than low-risk individuals? [More high risk] I think this point might be particularly important at the moment for countries such the European countries, where authorities try to vaccinate as much as possible their older population before moving on to vaccinate younger ones. Maybe the authors can discuss the extent to which their results can inform such strategies (and any other strategy currently used). Finally, the authors could add a note on what kind of data could be collected in order to assess for a given country or area which strategy to favor. I would, however, refrain from indicating exact values for homophily or correlation levels (as done in line 191), since this is a relatively simple model that most likely does not fit well reality.

We appreciate the reviewer's comment, and note that in the time since the comment was made many papers have appeared which explicitly address vaccine allocation strategies (Bubar et al 2021, Bubas et al 2021, Buckner et al 2021, Chen et al 2021, Ferranna et al 2021, Foy et al 2021, Islam et al 2021, Jentsch et al 2021, Matrajt et al 2021, Moore et al 2021). Our study is not intended to be a comprehensive investigation of optimal allocation strategies. Rather, we have highlighted certain results of our study that identify factors which might be important to developing an allocation strategy. The most prominent factor we identified as important for an appropriate vaccine allocation strategy is knowledge of the differential contact reduction practiced due to the presence of a pandemic by high-risk vs low-risk individuals. The original surveys that led to the creation of the age-age contact matrices were conducted prior to the pandemic, and data towards an equivalent age-age during-pandemic contact matrix, stratified ideally by comorbidity status (i.e., high-risk vs low-risk) would constitute a tremendous resource.

We have added clarifying text to the Methods sub-section "Effect of attribute values" (line 402) explaining in more detail how nodes become vaccinated in the model and hopefully clarifying that the prioritization is not meant (at least not directly) in a dynamic way:

> Once attribute values were assigned to each individual, each vaccinated person was removed from the pool of susceptibles with a probability corresponding to the considered vaccine effectiveness (that is, we consider an all-or-nothing vaccine). Moreover, note that as we study the spread and potential outbreak of an infectious disease in a local community, which typically happens within a few days to weeks, we do not consider vaccination as a dynamic process. In other words, the proportion

of vaccinated individuals is fixed for each simulation and vaccinations take place prior to the beginning of the simulation.

Figure 6 examines questions regarding the vaccine prioritization by comparing scenarios with positive (0.45), negative (-0.45) and no correlation between high-risk individuals and those who are vaccinated. The findings based on correlations imply that a higher proportion of high-risk than low-risk individuals should be vaccinated (assuming equal contact reduction, i.e., left side of plots). Similarly, the findings imply that knowledge of vaccination coverage only does not suffice to predict the severity of an outbreak but that who is vaccinated matters just like the homophily level among vaccinated individuals matters (Figure 2 & 3).

Finally, we agree with the reviewer that our results should be interpreted qualitatively rather than quantitively, and have reviewed the manuscript for instances where our language might be misleading. We have removed the language at line 184 indicated by the reviewer that quantifies the difference in the number of contacts between high-risk and low-risk individuals:

> If high-risk individuals have substantially fewer contacts (~~61-95% depending on the considered scenario;~~ Figure 6C), prioritizing the vaccination of low-risk individuals reduces overall mortality compared to homogeneous vaccination or vaccination of high-risk individuals.

Minor comments:
1. It could be good to give a clear definition of the important variables used in the paper early on in the text, especially for the outcome variables (e.g., the outbreak variable), and the terms used as labels in the figures (e.g., relative clustering). Again, the term clustering should be used with caution as it can refer to many things (structural clustering, attribute clustering, opinion clustering, etc.).

As discussed above (major comments 1 & 2), we have revised our language throughout the paper, replacing “clustering” and “relative clustering” with “homophily.” In addition, we have added a sentence to the Introduction (line 50) that defines “outbreak probability”:

> In the simple model, we focus on the probability of an infectious disease outbreak (defined as >1% becoming infected) as the primary outcome measure, while in the COVID-19 model, we focus on the number of deaths.

2. The second and sixth sentences of the abstract could be a bit clearer (in the second sentence, the “despite high vaccination coverage”, and in the sixth sentence the term “relative effects” could be better explained).

We have edited the abstract for clarification:

> …despite high population-wide vaccination ~~coverage~~ rates.

> In the presence of an effective vaccine, ~~relative~~ the effects of homophily and correlation of beliefs and circumstances become stronger.

3. In Figure 1, it is not clear what the authors mean by "removal of those successfully vaccinated". Also in the methods section, line 442, "recovered or dead individuals were removed from the simulations". Following the SIR terminology, my understanding is that they cannot get infected again, however, recovered and vaccinated people should still be potentially interacting with others and not fully removed from the social system. Is that the case? If not, it could be problematic for the interpretation of the model.

We thank the reviewer for identifying the need for clarification. To avoid future confusion, we included in the legend of Figure 1 a brief explanation relating to the all-or-nothing vaccine (i.e., either a vaccinated individual is protected following vaccination, or not. The latter happens with probability 1-vaccine effectiveness).

> (E)~Vaccination of all nodes with a positive (blue) attitude towards vaccines and removal of those successfully vaccinated (green); the probability that an all-or-nothing vaccine awards protection equals its effectiveness.

This explanation was thus far only found in the last paragraph of the Discussion and the Methods.

The reviewer is correct that vaccinated, dead, and recovered individuals are not removed from the social network, but they no longer affect the progression of the outbreak (i.e., for all practical purposes, they are "removed" from disease dynamics) because—in the model—they cannot be reinfected.

3. In Figure 1, it could be shown that the proportion of individuals who get vaccinated can change.

We only considered scenarios with a fixed vaccination coverage as we are investigating fast-paced disease outbreaks in local communities modeled using interaction networks. These outbreaks begin on the order of days to a few weeks, which is why we can treat the proportion of individuals who are vaccinated as fixed.

4. The differences between the first line and the second line of Figure 2 are not obvious to see. Maybe another visualization would help.

We thank the reviewer for pointing this out. We have revised Figure 2 in order to make the difference between 0% and 50% homophily more obvious (we now explicitly show the difference between the two scenarios), and have moved the original Figure 2 to the supplement.

5. In Figure 3 and 5, I personally find the 3-dimension correlation plots quite hard to read and interpret.

We sympathize with the reviewer that interpreting higher-dimensional plots can be difficult. The results of our study, however, are most salient in the higher-order interactions among the variables. Reporting that increased vaccine effectiveness reduces the probability of an outbreak is not very compelling, but reporting that the three-way interaction among the correlation of vaccinated and distancers, homophily of vaccinated, and vaccine effectiveness affects not only the strength but the direction of the effect on outbreak probability is highly salient. We have simplified Figure 3 (taken out a less important subplot), and understand that readers may need to take some time to fully understand the information being presented in these plots.

6. Regarding the choice of correlation values chosen for simulations, it does not seem so important to test correlations values with same magnitude and opposite signs. Based on what we know about the attributes under study and how they are distributed (i.e., correlations cannot be symmetrically distributed around zero), it seems more realistic to test correlation values ranging from zero (or low negative) to high.

We respectfully disagree with the reviewer. Disease dynamics change dramatically under a positive versus a negative correlation, as can be seen in the top row of (new) Figure 3A (old figure 3B): outbreak probability decreases with negative correlation but increases with positive correlation, holding other variables constant. We believe that reporting this result is informative as a counterexample to the current social situation, and helps elucidate disease dynamics. Furthermore, while it may be true that for the attributes under study a positive correlation is most realistic, the techniques developed in this study can be applied to a wide variety of situations, in some of which negative correlations among attributes might be expected. Ecological networks capturing competition among species within a guild is one example where we might expect negative correlations among attributes.

7. In Figure 6, it could help to show which regions of the plots correspond to the situation where we should vaccinate [high-]risk individuals in priority and to the other situation where we should vaccinate low-risk individuals in priority. This would really help the discussion on “switch points” for optimal strategies in the text, because it might be a bit hard to follow with the current figure.

We thank the reviewer for this suggestion, and have modified Figure 6 to include shaded areas indicating regions in which vaccinating low-risk or high-risk individuals is optimal.

8. The distinction between the model for the “generic infectious disease” and for Covid-19 is not obvious before reading the methods part (in particular, line 45 suggests that the only difference between the two models is the addition of high-risk individuals, which does not sound like a big difference at first sight). This being said, it is not because the model designed by the authors captures important features of the Covid-19 that it is necessarily an accurate representation of Covid-19 yet. For this reason, I would rephrase the sentence line 270.

We have clarified the text at line 47:

Then, in a more complex model developed specifically for COVID-19 (which includes hospitalization, asymptomatic carriers and differential risk status, McCombs and Kadelka 2020), we add a third binary attribute distinguishing between high- and low-risk individuals.

We also revised the sentence at line 294 in the Conclusion:

~~We studied the spread of a generic infectious disease as well as COVID-19 in interaction networks with various levels of homophily and correlations among beliefs and circumstances.~~

Using a simple generic infectious disease model as well as a more complex, previously developed model tailored specifically to COVID-19, we studied the spread of a virus in interaction networks with various levels of homophily and correlations among beliefs and circumstances.

9. I am not sure the term "social climate" is completely appropriate here and I wonder about its negative connotation. I would also keep in mind that homophily is probably not stronger nowadays than it was before, and the clustering of anti-vaxxers has certainly been around for a while.

We have revised this language to read "social context."

10. There is a typo line 166: "individual?s"

Thank you for pointing this out – it has been corrected.

11. The definition of the reduction of activity at the individual level (line 386) could be discussed. Right now two persons practicing social distancing are less likely to interact than two people with one social-distancer and one non-distancer. In reality, it could be that cautious individuals actively avoid others who are not being as careful as them, and prefer instead to interact with other cautious individuals (maybe practicing "social bubbles").

We thank the reviewer for identifying this issue, and have added the following paragraph to the Discussion (line 265):

In our model, two people who practice social distancing are less likely to interact than two people, one of whom practices social distancing and one of whom does not. This may not be a realistic assumption, as cautious individuals may avoid others who do not practice social distancing, and therefore interact more frequently with other social distancers (e.g., forming social bubbles). Detailed information regarding social distancing patterns under COVID-19 is currently lacking, however, and patterns may be heterogeneous across cultures and demographic groups. We therefore implemented a simple model for social interactions but acknowledge that our model could be extended to more realistic scenarios once more data become available.

12. It could be good to discuss how picking a seed node proportionally to its activity level affects the results of the model. I think the authors' results would be stronger if they could show similar dynamics for their model when the starting node is picked completely randomly.

We firmly believe that during a pandemic with nationwide social distancing efforts the most realistic modeling assumption regarding the seed case is that the social activity level is proportional to the probability that an individual starts/imports the disease to the local community being modeled. Future studies could compare the effect of the choice of seed case but we believe any other choice is less realistic.

13. I think it would be good to report here the different parameters for the transitions in the SIR model for high-risk individuals compared to low-risk (see line 440).

We thank the reviewer for pointing this out. We added the references to the specific tables in the prior publication, and added a sentence describing the risk-group-dependent parameters (line 469).

> We used the same probability distributions and parameters governing these transitions as in the original model description (Tables 1 and 2 in McCombs and Kadelka 2020). In particular, we assumed that high-risk individuals have a 1 to 5 times lower rate of truly asymptomatic infections, a 4 to 10 times higher hospitalization rate (when symptomatically infected), as well as a 4 to 10 times higher death rate (when hospitalized) (Verity et al 2020, Ferguson et al 2020).

14. There is a typo line 453: "th.0.e"

Thank you for pointing this out – it has been corrected.
